# Supplementary material for: Do Women Have a Higher Mortality Risk Than Men following ICU Admission after Out-of-Hospital Cardiac Arrest? A Retrospective Cohort Analysis
Source: J Clin Med. 2021 Sep 21;10(18):4286. doi: 10.3390/jcm10184286 (PMC8470772; doi:10.3390/jcm10184286)
Supplement: Supplementary file 1 [file jcm-10-04286-s001.zip › jcm-1336115-supplementary.pdf]

Table S1. Presenting the regression analyses only including ICU survivors

		Favourable CPC score (model 1)	ICU length of stay (model 2)
Men		1.341 (0.654 – 2.746)	0.685 (-0.505 - 1.875)
Age		**0.975 (0.952 – 0.997)**	-0.002 (-0.038 - 0.035)
Medical history	Hypercholesterolemia	1.272 (0.634 – 2.553)	-0.137 (-1.355 - 1.081)
	Former myocardial infarction	1.377 (0.596 – 3.179)	0.0553 (-0.963 - 2.069)
	Chronic lung disease	0.471 (0.152 – 1.463)	-0.137 (-1.774 - 1.499)
	Pulmonary embolism	2.667 (0.472 – 15.072)	-1.481 (-5.781 - 2.820)
	Former PCI	0.460 (0.162 – 1.305)	2.32 (-1.880 - 1.415)
	Former CABG	0.274 (0.059 – 1.278)	-0.396 (-2.463 - 1.670)
Medication use history	Aspirin	1.446 (0.634 – 3.300)	-0.707 (-2.214 -0.801)
	Calcium-channel blocker	1.125 (0.431 – 2.933)	0.862 (-0.958 - 2.682)
	Statins	0.753 (0.336 – 1.689)	-0.503 (-1.896 - 0.890)
Cardiac arrest characteristics	Location of arrest		
	Home	**0.476 (0.264 – 0.860)**	Reference
	Public	Reference	-0.662 (-1.644 - 0.320)
	Defibrillation by AED	0.860 (0.450 – 1.644)	0.034 (-1.069 - 1.138)
	Initial rhythm		
	Shock	Reference	-1.296 (-3.105 - 0.513)
	Non-shock	**0.310 (0.130 – 0.740)**	Reference
	Defibrillation by EMS	1.573 (0.717 – 3.452)	0.907 (-0.342 - 2.155)
	Cause of arrest		
	Cardiac	0.942 (0.185 – 4.809)	Reference
	Non-cardiac	Reference	1.834 (-0.987 - 4.655)
N		613	660
-2 Loglikelihood		361,678	
Likelihood ratio test p-value		0.017	
F-test (df; p-value)			0.813 (16,643; 0.671)
(Nagelkerke) R-square		0.101	0.02

Note: Estimates are Odds Ratios (OR), and beta's, and 95% Confidence Interval in parentheses (95% CI), derived from a Binary logistic regression analysis (model 1) and a linear regression analysis (model 2). CPC: Cerebral Performance Score, ICU: Intensive Care Unit, PCI: Percutaneous Coronary Intervention, CABG: Coronary Artery Bypass Graft, AED: Automated External Defibrillator, EMS: Emergency Medical Service. Bold values are significant at 5% alpha level.
